# Ribavirin inhibits peste des petits ruminants virus proliferation *in vitro*

**DOI:** 10.17221/56/2023-VETMED

**Published:** 2023-12-26

**Authors:** Weifeng Zhang, Hualong Deng, Yanfen Liu, Shaohong Chen, You Liu, Yuntao Zhao

**Affiliations:** ^1^Department of Animal Science, College of Coastal Agricultural Science, Guangdong Ocean University, Zhanjiang, P.R. China; ^2^Department of Bioengineering, College of Food Science and Technology, Guangdong Ocean University, Zhanjiang, P.R. China

**Keywords:** antiviral activity, antiviral drugs, mechanism, peste des petits ruminants, signal pathway

## Abstract

Peste des petits ruminants virus (PPRV), a member of the family Paramyxoviridae, belongs to the genus Morbillivirus. It causes devastating viral diseases in small ruminants and has been rapidly spreading over various regions in Africa, the Middle East, and Asia. Although vaccination is thought to be an effective management strategy against PPR infections, the heat sensitivity of PPRV vaccines severely restricts their use in regions with hot climates. In this research, we studied the antiviral activities of ribavirin and aimed to understand the potential mechanisms of action of ribavirin in the African green monkey kidney cells (Vero cells). In brief, the adsorption, intrusion, replication, and release of PPRV, as well as the mRNA expression level of RNA-dependent RNA polymerase (*RdRp*), were significantly inhibited in the ribavirin-treated Vero cells compared to those in the PPRV-infected cells that were not treated with ribavirin. Additionally, ribavirin has potential as an antiviral drug against PPRV, and its antiviral activity is mediated by the Janus kinase signal transducer and activator of transcription (JAK/STAT) and PI3K/AKT pathways.

Peste des petits ruminants virus (PPRV) causes an acute and highly infectious disease characterized by fever, mucopurulent ocular and nasal secretions, necrotising stomatitis, bronchopneumonia, and necrotising haemorrhagic enteritis in small ruminants, such as sheep and goats (Truong et al. 2014). The local sheep industry has suffered from enormous financial losses due to the rapid spread of the disease across many regions in Africa, the Middle East, and Asia, with morbidity and mortality rates reaching 100%. This has severely hampered the economic development of the sheep industry sector and endangered small ruminants (Pope et al. 2013; Pruvot et al. 2020). Therefore, the Food and Agriculture Organization of the United Nations (FAO) and the World Organization for Animal Health (WOAH) have developed a global strategy to control PPRV and eradicate its associated disease by 2030 (Njeumi et al. 2020).

PPRV is an important member of the genus *Morbillivirus* and the family *Paramyxoviridae*. This virus shares a close relationship with the measles, rinderpest, and canine distemper viruses (Barrett et al. 1993). Like the measles virus, PPRV is lymphocytophilic and causes immunosuppression in the host (Rajak et al. 2005). The non-structural protein C of the PPRV suppresses the retinoic acid-inducible gene I (RIG-I)- and melanoma differentiation-associated gene 5 (MDA5)-mediated interferon production by interacting with these proteins (Sanz Bernardo et al. 2017; Linjie et al. 2021). Interferon synthesis plays a crucial role in host cells’ innate defence against the virus. A decrease in interferon production due to viral infection and a weakened antiviral immune response may significantly cause immunosuppression in the host. Furthermore, the non-structural protein V, structural protein N, and P proteins inhibit interferon (IFN) and IFN-induced activation of the interferon-sensitive response element (ISRE) promoter, as well as activation of the interferon-γ-activated site (GAS) promoter in the JAK/STAT pathway, by preventing the phosphorylation and nuclear translocation of STAT1 and STAT2 (Chinnakannan et al. 2013; Ma et al. 2015; Li et al. 2019). Employing these mechanisms, the virus evades destruction by the host‘s innate immune system and induces immunosuppression, leading to secondary infections and complicating the diagnosis and treatment of the illness.

Vaccines are regarded as important tools for eliminating PPRV-induced disease. Live attenuated vaccines, such as Nigeria 75/1 and Sungri 96, have long been employed in disease prevention. To prevent the spread of diseases caused by PPRV in non-endemic regions, such as Europe, inactivated vaccines are still used instead of live attenuated vaccines because live attenuated vaccines are characterised by having low heat resistance and by their inability to distinguish between infected and vaccinated animals (Cosseddu et al. 2016).

To date, few *in* *vivo* or *in* *vitro* trials have screened antiviral medicines for diseases caused by PPRV, and no antiviral agents have been authorised for the treatment of PPR-induced viral diseases. Multiple PPRV outbreaks have occurred in some countries owing to the low vaccination coverage. Moreover, vaccination alone has proven inadequate for effectively managing PPRV outbreaks in nations with high vaccination rates. Additionally, the transmission of PPRV from domestic animals to wild small ruminants (Pruvot et al. 2020). This demonstrates that screening for antiviral medications against PPRV may offer early and efficient protection of the affected animals and help restrict the spread of the virus, which is of great value to the study field. Earlier *in vitro* studies (Lanave et al. 2017) showed that ribavirin, a drug with a broad antiviral spectrum, effectively prevents the replication of the canine distemper virus. This study aimed to evaluate the antiviral effectiveness of ribavirin against PPRV *in vitro* and conduct a thorough analysis of its mechanism of action.

## MATERIAL AND METHODS

### Cells and virus

Vero cells were cultured in Dulbecco’s Modified Eagle’s Medium (DMEM; Gibco, Grand Island, NY, USA) containing 10% foetal bovine serum (FBS; Gibco, Grand Island, NY, USA) with penicillin (100 U/ml) and streptomycin (100 μg/ml) (Sigma-Aldrich, St. Louis, MO, USA). In this study, the PPRV vaccine strain Nigeria 75/1 (China Institute of Veterinary Drug Control, Beijing, P.R. China) was used to infect Vero cells.

### Antibodies and drugs

Details of the antibodies and drugs are provided below (Table 1). The polyclonal antibodies against the nucleocapsid (N), fusion (F), and haemagglutinin (H) proteins of PPRV used in this study were developed as described previously.

**Table 1:** Antibodies and drugs

Antibodies	Source	Identifier
Bcl-2-associated agonist of cell death (BAD)	Santa Cruz Biotechnology	Cat# 8044
phosphorylated (p)-BAD	Santa Cruz Biotechnology	Cat# 271963
B-cell lymphoma 2 (Bcl-2)	Santa Cruz Biotechnology	Cat# 7382
cAMP response element binding protein (CREB)	Santa Cruz Biotechnology	Cat# 377154
phosphorylated (p)-CREB	Santa Cruz Biotechnology	Cat# 81486
Glycogen synthase kinase 3 (GSK3)	Santa Cruz Biotechnology	Cat# 7291
phosphorylated (p)-GSK3	Santa Cruz Biotechnology	Cat# 3738
Janus kinase 1 (JAK1)	Cell Signaling Technology	Cat# 3716
Signal transducer and activator of transcription 1 (STAT1)	Cell Signaling Technology	Cat# 6772
phosphorylated (p)-STAT1	Cell Signaling Technology	Cat# 6772
Phosphatidylinositol 3-kinase (PI3K)	Cell Signaling Technology	Cat# 4249
Protein kinase B (AKT)	Cell Signaling Technology	Cat# 4691
phosphorylated (p)-AKT	Cell Signaling Technology	Cat# 4060
Nuclear factor kappa B (NF-κB) p65	Cell Signaling Technology	Cat# 6956
phosphorylated (p)-NF-κB p65	Cell Signaling Technology	Cat# 3033
		
Drugs		
Ribavirin	Aladdin Bio-Chem Technology Co	Cat# R101754

### Assessment of ribavirin cytotoxicity

The cytotoxicity of ribavirin was measured using the MTT assay (Sen et al. 2010). In brief, Vero cells were inoculated at 1 × 10^4^ cells/well in 96-well plates and incubated at 37 °C and 5% CO_2_. The cells were grown for 48 h at 37 °C with varying ribavirin concentrations (two-fold dilutions from the starting concentration of 100 μg/ml). After adding 20 μl of the MTT reagent to each well, the cells were incubated at 37 °C for an additional 4 hours. The cells were then treated with 150 μl DMSO for 10 min with shaking to achieve solubilisation. Finally, an ELISA microplate reader (BioTek, Winooski, VT, USA) was used to measure the optical density of each well at 490 nm.

### Antiviral effect of ribavirin

Vero cells were grown in 96-well plates at a density of 1 × 10^4^ cells per well at 37 °C and 5% CO_2_. Ribavirin, at the highest noncytotoxic concentration, and a multiplicity of infection (MOI) of 7 of PPRV were concurrently introduced into each well after medium removal, and the plate was then incubated for 48 h at 37 °C. The controls included both cellular and PPRV-infected cells (Yun et al. 2021).

### Immunofluorescence assay (IFA)

On the coverslips positioned at the bottom of each well of a six-well plate, Vero cells were sown and cultured at 37 °C until they reached confluency. Cells processed with PPRV alone acted as the virus control group, while those treated with a combination of ribavirin and PPRV were cultured at 37 °C and 5% CO_2_. The cells were then removed, fixed in 4% paraformaldehyde for 15 min, permeabilised with phosphate-buffered saline (PBS) containing 0.1% Triton X-100 for 15 min, blocked with 5% bovine serum albumin (BSA) for 1 h, incubated with anti-PPRV serum overnight at 4 °C, and then incubated with fluorescein isothiocyanate (FITC)-conjugated secondary antibodies for 1 h at 25 °C. Finally, the cell nuclei were counterstained with 4',6-diamidino-2-phenylindole (DAPI) and observed using a fluorescence microscope (Leica, Wetzlar, Germany), as previously reported (Im et al. 2019).

### Western blotting

Vero cells were placed in cell culture flasks and incubated at 37 °C and 5% CO_2_ until they were confluent to observe the effectiveness of ribavirin on PPRV. The cells were subsequently infected with an MOI of 7 of PPRV at a final dosage of 12.5 μg/ml ribavirin, while the cells handled with PPRV alone served as a pure viral control. After a 50% cytopathic effect (CPE) was observed in the virus control group, proteins were extracted from the harvested cells using RIPA buffer (Beyotime, Shanghai, P.R. China). Protein concentrations were determined using a bicinchoninic acid assay (BCA). Protein was then separated by 10% SDS-PAGE, transferred onto a polyvinylidene fluoride (PVDF, Millipore, Billerica, MA, USA) membrane, blocked with 5% skimmed milk for 1 h, incubated overnight at 4 °C with the primary antibody, followed by a 1-hour incubation with horseradish peroxidase (HRP)-conjugated secondary antibody. Bands were visualised using chemiluminescence, as previously reported (Liu et al. 2018).

### Quantitative real-time reverse transcription–polymerase chain reaction (qRT-PCR)

Ribavirin at a final concentration of 12.5 μg/ml and PPRV (MOI of 7) was used to treat the Vero cells at 60% confluence for 48 hours. Total RNA was extracted from the cells using TRIzol reagent (Trans Gen, Beijing, P.R. China), and the concentrations were determined with microplate photometers (Bio Tek). Subsequently, 3 μg of the extracted RNA was reverse transcribed using the All-In-One RT Super Mix Kit (Vazyme, Nanjing, P.R. China), following the manufacturer‘s instructions. The ChamQ Universal SYBR qPCR Master Mix (Vazyme, Nanjing, P.R. China) was used for the qPCR reactions. mRNA expression levels of the target gene were calculated using the 2^−ΔΔCT^ method, with *GAPDH* serving as the housekeeping gene, as shown in Table 2.

**Table 2:** Primer sequences for qRT-PCR

Gene name	Sense-strand (5'-3')	Anti-sense strand (5'-3')
*PPRV N*	CTCGGAAATCGCACAGAC	TCTTCTCTGGTCGCTGGT
*PPRV H*	ATGGTTGTATTGCCGACGAAGGAC	GAGGAACTTAATCTTATCG
*RdRp*	AGGGATGCTGCTCGGTCTTGG	CGTGTAGGTGTAGCACTGTGG
*PKR*	ATAGCAAGAAGGCAGAGCGTGAAG	TCAAGTCCATCCCAACAGCCATTG
*IRF9*	GACTACTCACTGCTGCTCACCTTC	GCTCCATGCTGCTCTCAGAACC
*ISG20*	ATGGACTGCGAGATGGTGGG	CCCTCAGGCCGGATGAACTT
*ISG54*	CCAACCAAGCAAGTGTGAGGAGTC	CTTCTGCCAGTCTGCCCATGTG
*MxA*	GCATCTCCAGCCACATCCCTTTG	TGGTGTCGCTCCGCTCCTTC
*GADPH*	CATGACCACAGTCCACGCCATC	GATGACCTTGCCCACAGCCTTG

### Impact of ribavirin on PPRV adhesion to Vero cells

The impact of ribavirin on PPRV adhesion to Vero cells was assessed using IFA and qRT-PCR. Vero cells were seeded on coverslips, exposed to PPRV and ribavirin, and then analysed using IFA, as described previously (Zhang et al. 2021).

The cells were infected with MOI of 7 of PPRV at 4 °C for 2 h after pre-treatment with ribavirin at 37 °C for 1 hours. After harvesting the cells and washing them five times with PBS, qRT-PCR was performed to calculate the expression of the PPRV gene.

### Impact of ribavirin on PPRV entry into Vero cells

Vero cells were infected with PPRV at an MOI of 7 in a six-well plate at 4 °C for 1 h, washed three times with cold PBS, treated with ribavirin at 37 °C for 1 h, re-washed with PBS to remove any extracellular viral particles, and then incubated with DMEM containing 2% FBS for 36, 48, and 60 h, as mentioned above (Khandelwal et al. 2014). Western blotting was performed to determine the presence of the N, F, and H structural proteins of PPRV in the cell lysates.

### Statistical analysis

The results are expressed as the mean ± standard deviation and were analysed using GraphPad Prism 5 (GraphPad Prism Software, La Jolla, CA, USA).

Student’s *t*-test was used to analyse the differences between the two groups, while one-way analysis of variance (ANOVA) was used to compare the differences between numerous groups.

Statistical significance was set at a *P*-value < 0.05.

## RESULTS

### Cytotoxic effects of ribavirin on Vero cells

**Figure 1:**
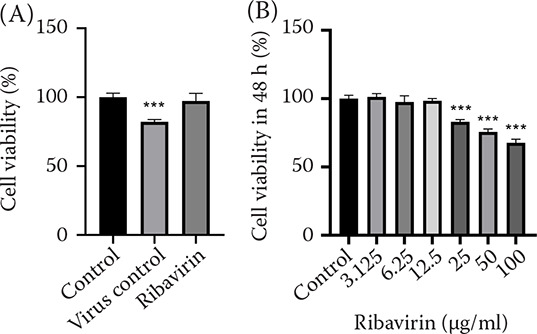
Identifying the maximum noncytotoxic concentration of ribavirin (A) The vitality of Vero cells after being treated with ribavirin serially diluted over the course of 48 h from a starting concentration of 100 μg/ml. (B) The viability of Vero cells was treated for 48 h with MOI of 7 of PPRV and the highest permissible dosage of ribavirin. The data, obtained from three independent experiments, are presented as the mean ± SD (***P < 0.001 vs the cell control)

The cytotoxic effect of ribavirin on Vero cells was determined using MTT assay. The maximum non-toxic concentration of ribavirin was 12.5 μg/ml (Figure 1A).

### Antiviral effectiveness of ribavirin against PPRV *in vitro*

The viability of the ribavirin-treated group was significantly higher than that of the virus control group, and no significant lesions were observed (Figure 1B).

**Figure 2:**
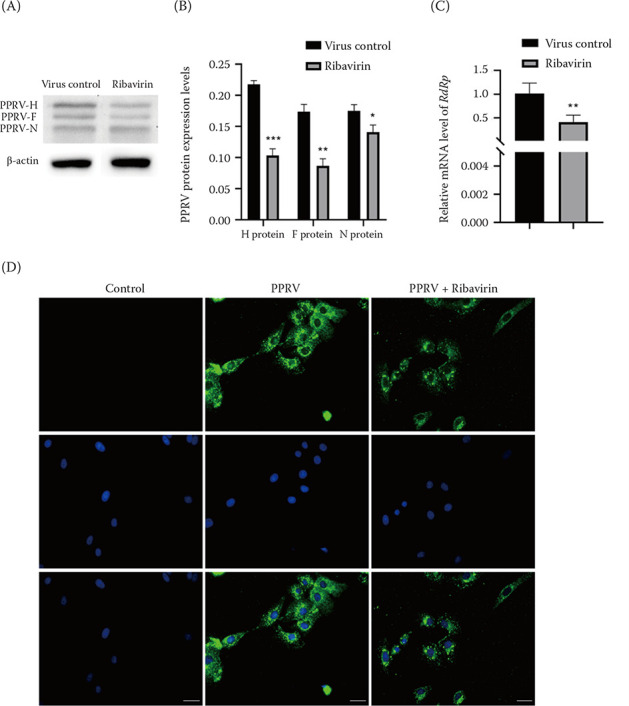
The impact of ribavirin on PPRV in Vero cells (A,B) PPRV (MOI of 7) and 12.5 μg/ml of ribavirin were administered to Vero cells for 48 h, after which the expression of the structural proteins H, F, and N was examined by western blotting. The data, collected from three independent experiments, are presented as the mean ± SD (*P < 0.05, ** *P* < 0.01, *** *P* < 0.001 vs the virus control). (C) For the purpose of measuring the relative mRNA expression levels of the *RdRp*, Vero cells were concurrently treated with ribavirin and PPRV for 48 h (**P < 0.01 vs the virus control). (D) After 48 h, IFA was used to identify the anti-PPRV activity of ribavirin in the virus control group. PPRV is stained in green, and the nuclei are stained in blue (DAPI). Scale bars = 50 μm

According to the western blot analysis (Fig-ure 2A,B), the expression levels of PPRV proteins N, F, and H were significantly decreased in ribavirin-treated cells relative to the viral control cells. Furthermore, the qRT-PCR results demonstrated that ribavirin treatment dramatically lowered the *RdRp* expression as measured (Figure 2C). In addition, IFA analysis showed a significant reduction in the viral particles dispersed in the cytoplasm in ribavirin-treated Vero cells compared with those in the virus control. Ribavirin-treated cells displayed fewer syncytia than those observed in non-ribavirin-treated PPRV-infected cells. This implies that ribavirin prevented the replication of PPRV in Vero cells and rendered the viral infection less harmful to the cells (Figure 2D).

### Influence of ribavirin on various stages of the PPRV infection cycle

**Figure 3:**
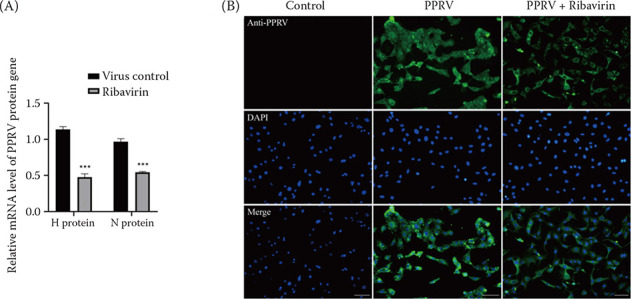
The effect of ribavirin on PPRV attachment to Vero cells Influence of ribavirin on the capability of PPRV to attach to the host cells Vero cells were first pre-incubated with 12.5 μg/ml of ribavirin or an equal volume of media at 37 °C for 1 h, and then PPRV was infected for 2 h at 4 °C. The cells were washed three times with cold PBS for qPCR and IFA detection. The data, obtained from three independent experiments, are presented as the mean ± SD (***P < 0.001 vs the virus control) (A) The mRNA expression level of the *H* and *N* genes of PPRV. (B) Indirect IFA was used to identify the PPRV distribution in the cytoplasm. Scale bars = 100 μm

The PPRV *H* and *N* gene expression levels were considerably decreased in ribavirin-treated Vero cells compared to the viral controls (Figure 3A). According to the IFA data, the quantity of PPRV particles scattered in the cytoplasm was dramatically decreased following ribavirin treatment, and most infected cells had normal morphology. However, in the untreated control group, round and merged cells were observed (Figure 3B). These findings imply that ribavirin dramatically reduced the PPRV adhesion to Vero cells.

**Figure 4:**
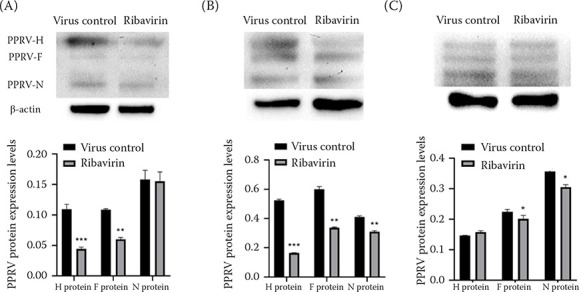
The impact of ribavirin on the expression levels of PPRV structural proteins H, F and N After infecting Vero cells with MOI of 7 of PPRV at 4 °C for 1 h, the unattached virus was washed with pre-cooled PBS. The cells were then treated with 12.5 μg/ml of ribavirin at 37 °C for 1 hours. Finally, the cells were washed with PBS replaced with DMEM containing 2% FBS, and incubated at 37 °C. The cells were collected after (A) 36, (B) 48, and (C) 60 h of invasion, and the expression of viral structural proteins H, F, and N was analysed by western blotting. The data, obtained from three independent experiments, are presented as the mean ± SD (*P < 0.05, ** *P* < 0.01, *** *P* < 0.001 vs the virus control)

The expression levels of the three PPRV structural proteins were significantly reduced in the ribavirin-treated Vero cells (H protein at 36 and 48 h; F protein at 36, 48, and 60 h; and N protein at 48 and 60 h post-intrusion) (Figure 4). This indicated that ribavirin treatment obstructed PPRV intrusion.

Furthermore, expression levels of the aforementioned PPRV structural proteins increased markedly with intrusion time, approaching those observed in the virus control group. This suggests that early ribavirin treatment in the infection cycle can prevent PPRV from entering the host cells.

### Ribavirin’s anti-PPRV activities are mediated by the PI3K/AKT and JAK/STAT signalling pathways

**Figure 5:**
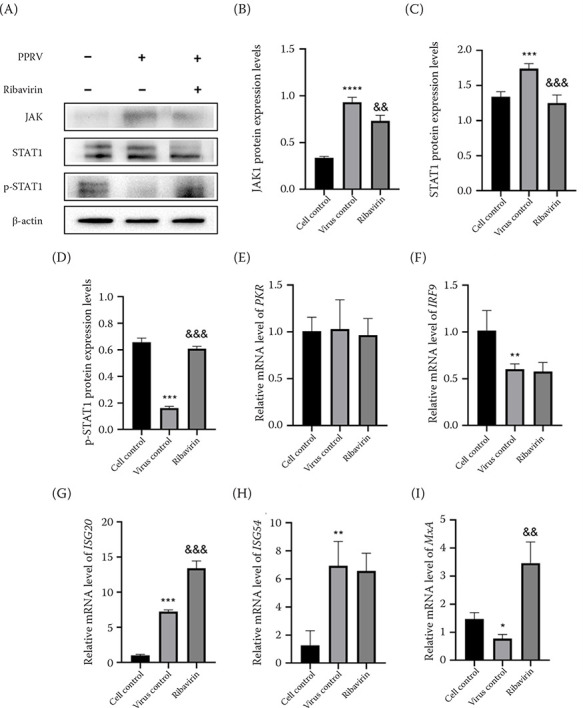
The impact of ribavirin on the JAK/STAT signalling pathway’s associated expression levels of proteins and mRNAs (A–I). Vero cells were infected with PPRV and treated with ribavirin at the same time. Protein expression levels were then measured 48 h later. JAK1 (B), STAT1 (C), and p-STAT1 (D) expression levels were determined by western blotting. qRT-PCR was used to identify the relative mRNA levels of the following genes: *PKR* (E), *IRF9* (F), *ISG20* (G), *ISG54* (H), and *MxA* (I). The data, derived from three independent experiments, are presented as the mean ± SD (&&*P* < 0.01, &&& *P* < 0.001 vs the virus control; * *P* < 0.05, ** *P* < 0.01, *** *P* < 0.001 vs the cell control)

To further understand the molecular mechanisms underlying the antiviral actions of ribavirin, we measured the expression levels of key proteins implicated in the JAK/STAT and PI3K/AKT signalling pathways. The expression levels of proteins STAT1 and JAK1 were considerably lower, whereas those of p-STAT1 were noticeably higher in PPRV-infected cells treated with ribavirin than in the virus-control cells (Figure 5A–D). This suggests that the JAK/STAT signalling pathway was attenuated in PPRV-infected cells and that ribavirin treatment may potentiate it.

The mRNA expression levels of the interferon regulatory factor 9 (*IRF9*), myxovirus resistance protein A (*MxA*), interferon-stimulated gene 20 (*ISG20*), IFN-stimulated gene 54 (*ISG54*), and double-stranded RNA-dependent protein kinase (*PKR*) are all associated with the innate antiviral activity in the host cells. Following the PPRV infection of the Vero cells, the mRNA expression levels of *ISG20* and *ISG54* were substantially upregulated, whereas the expression levels of *MxA* and *IRF9* were significantly reduced, and *PKR* expression levels were unaffected compared with their levels in cell control. Compared with their levels in the viral-infected control cells, ribavirin therapy significantly increased the *ISG20* and *MxA* expression levels in the PPRV-infected cells (Figure 5E–I).

**Figure 6:**
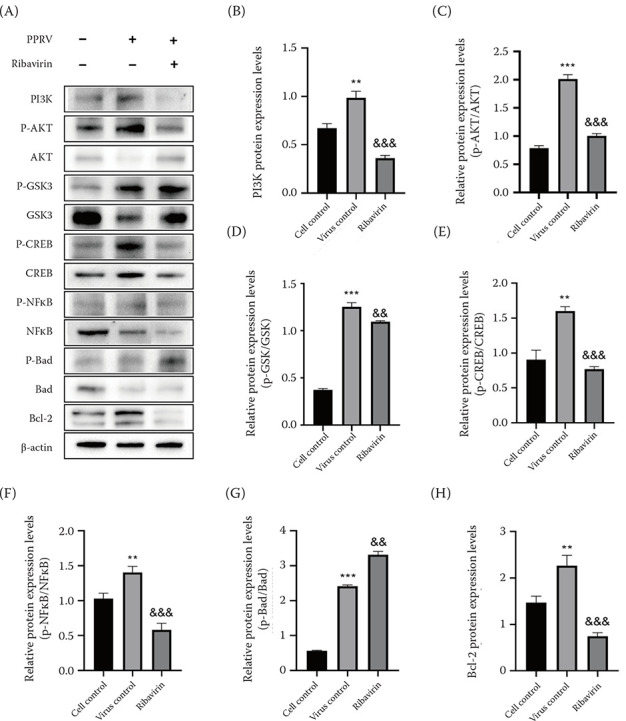
The impact of ribavirin on the expression levels of the essential proteins of the PI3K/AKT signalling pathway Vero cells were treated with ribavirin and infected with PPRV, and the expression levels of PI3K, p-AKT/AKT, p-GSK/GSK, p-CREB/CREB, p-NF-κB p65/NF-κB p65, p-Bad/Bad, and Bcl-2 (A–H) were identified by western blotting 48 h later. The data, derived from three independent experiments, are presented as the mean ± SD (&&&*P* < 0.001 vs the virus control; ** *P* < 0.01, *** *P* < 0.001 vs the cell control)

PI3K and p-AKT [Electronic Supplementary Material (ESM): Figure S1B] expression levels were dramatically increased, the p-AKT/AKT ratio was upregulated, and the AKT (ESM Figure S1A) expression level was decreased in the virus control group compared to cellular controls (untreated and uninfected). Ribavirin treatment substantially decreased both PI3K expression and the p-AKT/AKT ratio compared to the viral control group (Figure 6B,C).

The GSK3 (ESM Figure S1C) expression level was marginally downregulated in the virus control cells, whereas the p-GSK3 (ESM Figure S1D) expression level was upregulated compared to that in the cell control. Additionally, compared with those in the viral control, ribavirin lowered the expression levels of CREB, p-CREB (ESM Figure S1E,F), and Bcl-2 (Figure 6A,H). Moreover, ribavirin significantly reduced the ratios of p-NF-B p65/NF-B p65, p-CREB/CREB, and p-GSK3/GSK3 compared to those in the viral control (Figure 6D–F). Notably, the BAD (ESM Figure S1G) expression level was significantly decreased, and the ratio of p-BAD/BAD was increased in the PPRV-infected cells compared to those in the cell control (Figure 6G). Furthermore, the p-BAD (ESM Figure S1H) expression level and p-BAD/BAD ratio significantly increased after ribavirin treatment compared to those before ribavirin treatment.

These findings suggest that the antiviral activity of ribavirin is mediated through the PI3K/AKT and JAK/STAT signalling pathways. Ribavirin treatment reduced the PPRV-induced activation of the PI3K/AKT signalling pathway and dramatically affected the expression levels of the downstream signalling molecules related to apoptosis control, thereby blocking PPRV replication in the host cells. Conversely, PPRV infection suppressed the JAK/STAT signalling pathway, ribavirin treatment reversed this effect by increasing p-STAT1 expression, and boosting the expression levels of mRNAs *MxA* and *ISG20*, resulting in improved innate immunity.

## DISCUSSION

Ribavirin is a nucleoside antiviral drug that was discovered in 1972 as a guanosine analogue. Ribavirin has broad-spectrum anti-RNA and DNA viral activity and can be used to treat infections caused by Respiratory syncytial virus, Newcastle disease virus, influenza virus, canine distemper virus, and PPRV (Naik and Tyagi 2012). Previous studies have focused on the antiviral mechanisms of ribavirin. Ribavirin monophosphate, a phosphorylation product of ribavirin, may be structurally similar to inosine monophosphate (IMP), which inhibits the viral RNA replication by affecting the binding of hypoxanthine nucleotides to inosine-5‘-monophosphate dehydrogenase (IMPDH) (Parker 2005), which may, in turn, inhibit the RdRp activity, thereby inhibiting viral replication. Since the nucleotide analogues of the RNA polymerase reaction substrates of drugs are comparable, they are more likely to affect the efficiency of the viral RNA polymerase synthesis and exhibit antiviral effects (Eriksson et al. 1977). Moreover, the triphosphate derivatives of ribavirin may act as a mutagen, generating base mismatches during RNA virus replication (Crotty et al. 2000). In addition, ribavirin can enhance the cellular immune response and exert antiviral effects (Lanford et al. 2001). However, the anti-PPRV activity of ribavirin and its associated mechanism of action remains unclear.

In this study, we determined that ribavirin inhibits PPRV replication and the CPE of PPRV infection. According to our findings, ribavirin suppresses viral adsorption, intrusion, replication, and release. Recent studies have shown that epigallocatechin gallate, a primary component of green tea, directly binds to PPRV particles, exhibiting antiviral effects (Saadh 2021). Furthermore, buquina (BQR) and leflunomide (LFM), two specific inhibitors of dihydroorotate dehydrogenase (DHODH), and 6-azidouridine (6-AU), an inhibitor of orotide-5'-monophosphate decarboxylase (ODase), inhibit PPRV replication *in vitro* by depleting purine nucleotides, which can also trigger the transcription of antiviral interferon-stimulated genes (ISG) (Jin et al. 2021). Ribavirin significantly inhibits PPRV replication by inhibiting IMPDH (Chang et al. 2018); however, the mechanism by which ribavirin inhibits PPRV infection has not been investigated. In our study, ribavirin was observed to significantly inhibit the expression of PPRV RdRp and suppress PPRV intrusion and release, suggesting that ribavirin may interdict interactions between PPRV envelope glycoproteins (N and H) and the receptors of host cells. Therefore, we hypothesised that antiviral drugs may have different mechanisms of action.

Natural immunity is the first line of defence against foreign invasion in the body. Interferons affect the JAK/STAT pathway during the viral infection, eventually increasing the expression of ISG-associated antiviral proteins (Hao et al. 2014). The expression of the antiviral proteins inhibits viral invasion and replication, induces apoptosis in the infected cells, and destroys the viral RNA (Shaposhnikov et al. 2013). Interferon activation of the JAK/STAT signalling pathway is a crucial mechanism by which the host cells combat the viral infection. Therefore, viruses have developed different ways to circumvent the interferon-mediated antiviral defence of host cells. A previous study has shown that the C and V proteins encoded by the measles virus can bind to IFN receptors on the host cells, causing the IFN receptor complex to malfunction (Yokota et al. 2003). Additionally, JAK1 and STAT may interact and inhibit phosphorylation by the measles virus V protein (Caignard et al. 2007). PPRV targets JAK/STAT signalling through an array of mechanisms. On one hand, the N protein of PPRV can inhibit the nuclear translocation of IRF3 and prevent interferon release from the host cell. Moreover, the N protein may inhibit the expression of antiviral genes linked to ISGs (Zhu et al. 2019). Finally, N and P proteins block STAT1 phosphorylation by binding to p-STAT1 (Li et al. 2019).

In this research, JAK/STAT signalling was suppressed in the virus control cells; however, p-STAT1 levels increased significantly following ribavirin treatment, suggesting that ribavirin may reactivate the JAK/STAT system. Thus, the mRNA expression levels of the downstream signalling molecules linked to the JAK/STAT pathway of innate antiviral immunity, ISG20 and MxA, were considerably increased in the PPRV-infected and ribavirin-treated cells compared to those in the viral control. These findings suggest that ribavirin may use the JAK/STAT pathway to modulate the immunological response of the host cells against PPRV.

The PI3K/AKT signalling pathway is a common apoptotic signalling pathway. A growing number of studies have shown that viruses may employ the PI3K/AKT signalling pathway to activate downstream signalling molecules, thus extending cell survival and facilitating their own replication and proliferation. For instance, herpes simplex virus type 1 (HSV-1) infection of the host cells activates the PI3K/AKT signalling pathway, whereas sophoridine (SRI) treatment significantly reduces AKT phosphorylation levels and inhibits HSV-1-induced activation of the PI3K/AKT signalling pathway, thereby inhibiting HSV-1 replication (Tang et al. 2022). Moreover, measles virus infection of the host cells decreases AKT protein activity (Avota et al. 2001). Numerous studies have shown that viruses may exploit the PI3K/AKT signalling pathway to enable better replication and proliferation. However, the impact of PPRV on the PI3K/AKT signalling system remains unknown. According to current research, ribavirin treatment inhibits PI3K/AKT signalling pathway activation induced by PPRV infection.

Changes in the downstream expression of proteins in the PI3K/AKT pathway, such as GSK3, Bcl-2, CREB, BAD, and NF-κB, are intimately related to cell proliferation, apoptosis, and immunological modulation (Beurel et al. 2010). We found that PPRV significantly altered the expression levels of several downstream signalling molecules in the PI3K/AKT pathway. PPRV infection of the host cells dramatically increased the p-AKT and PI3K expression levels, and considerably enhanced the p-AKT/AKT ratio. The downregulation of p-AKT and PI3K expression in the PPRV-infected cells after ribavirin treatment suggests that ribavirin may prevent the PPRV-mediated activation of the PI3K/AKT signalling pathway. Additionally, the expression levels of p-GSK3, Bcl-2, p-NF-B p65, p-CREB, and CREB were dramatically downregulated after ribavirin treatment compared to those in the untreated PPRV-infected cells. Simultaneously, the ratios of p-NF-B p65/NF-B p65, p-CREB/CREB, and p-GSK3/GSK3 decreased considerably.

Ribavirin decreased the growth of PPRV in the viral-infected cells *in* *vitro* and diminished the cytopathic lesions generated by PPRV. Ribavirin treatment upregulates the phosphorylation of STAT1 and defunds the inhibition of the JAK/STAT signalling pathway induced by PPRV infection. Additionally, ribavirin treatment upregulates the mRNA expression of ISG20 and MxA, which are signalling molecules downstream of the JAK/STAT signalling pathway, thereby enhancing the antiviral immune response of the host cells. In contrast, ribavirin treatment suppressed the activation of the PI3K/AKT signalling pathway, regulated the expression levels of the signalling molecules downstream of the PI3K/AKT pathway, triggered apoptosis in PPRV-infected cells, and prevented the proliferation of PPRV in the host cells. Consequently, these findings indicate that ribavirin may be a promising therapeutic drug for PPR.
