# Supplementary material for: Ribavirin inhibits peste des petits ruminants virus proliferation in vitro
Source: Vet Med (Praha). 2023 Dec 26;68(12):464–76. doi: 10.17221/56/2023-VETMED (PMC10828777; doi:10.17221/56/2023-VETMED)
Supplement: Supplementary Figure 1 [file VETMED-68-12-123056-s001.pdf]

https://doi.org/10.17221/56/2023-VETMED

Ribavirin inhibits peste des petits ruminants virus proliferation *in vitro*

Weifeng Zhang[1], Hualong Deng[1], Yanfen Liu[1], Shaohong Chen[2], You Liu[2], Yuntao Zhao[2]**

[1]*Department of Animal Science, College of Coastal Agricultural Science, Guangdong Ocean University, Zhanjiang, P.R. China*
[2]*Department of Bioengineering, College of Food Science and Technology, Guangdong Ocean University, Zhanjiang, P.R. China*

**Corresponding authors: liuy6254282@163.com; yuntaozhao@163.com*

Weifeng Zhang and Hualong Deng contributed equally to this work

The authors are fully responsible for both the content and the formal aspects of the electronic supplementary material. No editorial adjustments were made.

Electronic Supplementary Material (ESM)

Figure S1. The impact of ribavirin on the expression levels of the essential proteins of the PI3K/AKT signalling pathway

https://doi.org/10.17221/56/2023-VETMED

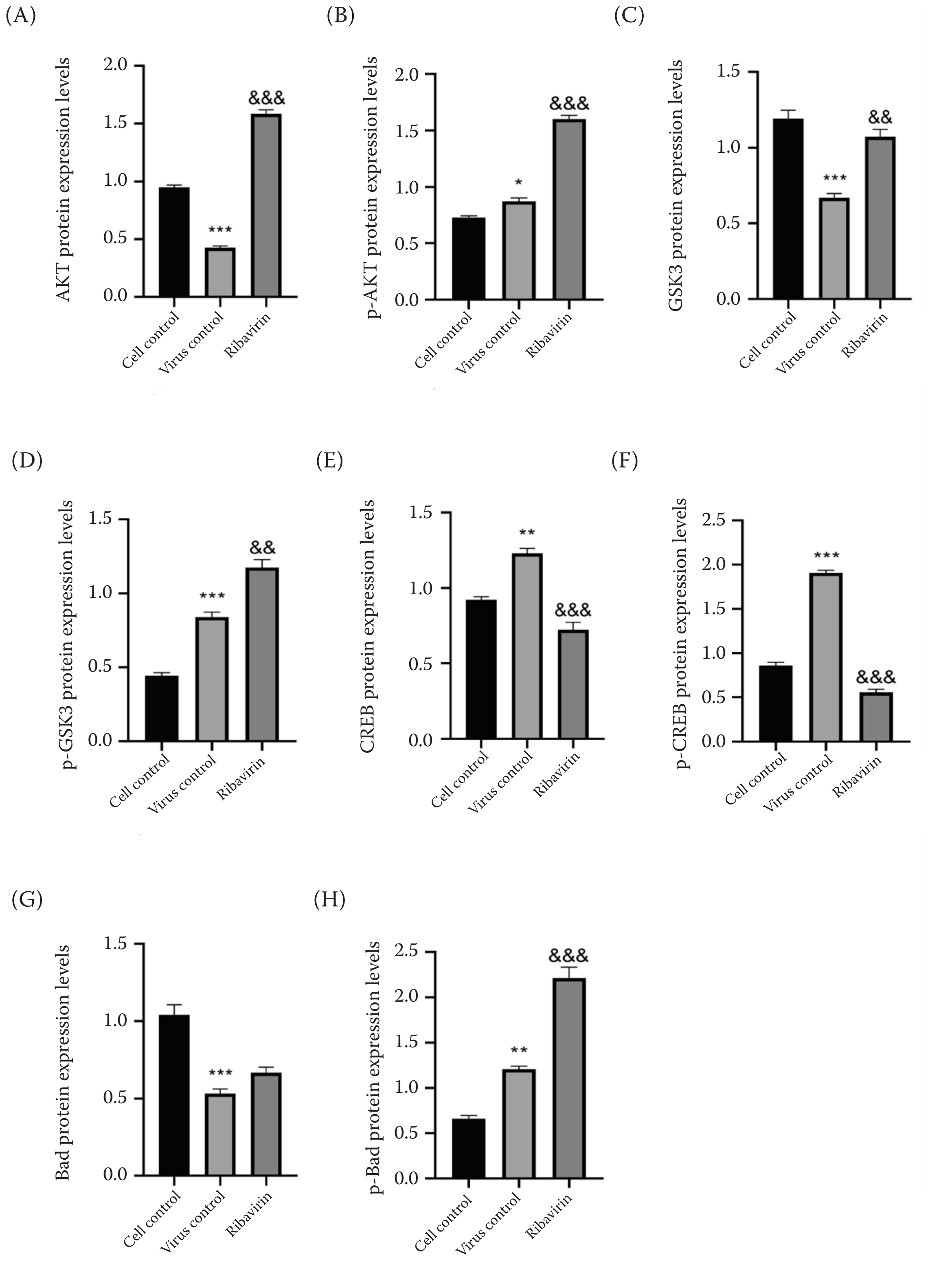

Figure S1. The impact of ribavirin on the expression levels of the essential proteins of the PI3K/AKT signalling pathway Vero cells were treated with ribavirin and infected with PPRV, and the expression levels of AKT, p-AKT, GSK3, p-GSK3, CREB, p-CREB, Bad, and p-Bad (A–H) were identified by western blotting 48 hours later. The data, obtained from three independent experiments, are presented as the mean ± SD (&&$P < 0.01$, &&& $P < 0.001$ vs the virus control; *$P < 0.05$, **$P < 0.01$, ***$P < 0.001$ vs the cell control)
